# Supplementary material for: Personalized music for cognitive and psychological symptom management during mechanical ventilation in critical care: A qualitative analysis
Source: PLoS One. 2024 Oct 24;19(10):e0312175. doi: 10.1371/journal.pone.0312175 (PMC11500878; doi:10.1371/journal.pone.0312175)
Supplement: S1 File — (DOCX) [file pone.0312175.s001.docx]

**S1: Interview Guide**

1. Tell me how you came to choose the music you’ve been listening to/we’ve been playing for [insert loved one’s name]?

2. Describe some of the times you/[insert participant’s name] listen(s) to or play music at home? In life?

(*Probe: describe some different scenarios when they listen to, play, or use music in their lives.*

*Probe: describe their relationship with music*

*Probes: Do they play an instrument/DJ, do they work with music or study it?)*

3. How would you describe the music you chose?

4. What do you think is going through [insert loved one’s name]’s mind right now?

5. What sort of symptoms or feelings have you experienced/do you think [insert name] is experiencing?

*Probe: Have you had pain or discomfort?/Do you think they are uncomfortable?*

6. How have you been affected by music/do you think this music will/has affected [insert name]? Physically, emotionally?

7. And how do you think music works on people?

8. How does the music experience change between the hospital and home?

9. How has music influenced your views of this hospitalization?

10. How, if at all, has having a music choice influenced your views of this hospitalization?

11.If you had to rate the music you’ve chosen on a scale of 1-5 with 5 being very personal, and 1 being not so personal, how personal is this music selection to you/[insert participant];

12. What about familiar? How familiar is this music to you/[insert participant] Same scale, is it extremely familiar or not so much?

13. And, what about preferred where 5 is all the way you/their favorite and 1 is not liked at all.

14. How do you feel about music? Can you describe your own experiences and history with music?

15. What is one thing you want to tell me about the study of music and being in the hospital?
