# Supplementary material for: Personalized music for cognitive and psychological symptom management during mechanical ventilation in critical care: A qualitative analysis
Source: PLoS One. 2024 Oct 24;19(10):e0312175. doi: 10.1371/journal.pone.0312175 (PMC11500878; doi:10.1371/journal.pone.0312175)
Supplement: S2 File — (DOCX) [file pone.0312175.s002.docx]

S2: Other Informants

Mother of young man with coma after motor vehicle crash (MVC)

Mother of young man with coma after pedestrian versus automobile (PVA)

Partner of young man with traumatic brain injury (TBI) after bicycle crash

Sister of middle-aged man with coma after MVC and cerebral vascular accident (CVA)

Middle aged man with blunt chest trauma

Bedside registered nurses

Sister of young woman with TBI after MVC

Medical doctors of neurology, critical care, surgery and neurosurgery: professors, and trainees

Wife and sister-in-law of man with TBI

Middle aged woman after abdominal surgery

Middle aged man with abdominal injuries

Family of older woman after CVA

Advanced Practice Providers on the trauma service

Uncle and father of young man with assault related injuries

Parents and sister of young man with TBI and assault

Siblings of young man with assault related injuries
