# Supplementary material for: Personalized music for cognitive and psychological symptom management during mechanical ventilation in critical care: A qualitative analysis
Source: PLoS One. 2024 Oct 24;19(10):e0312175. doi: 10.1371/journal.pone.0312175 (PMC11500878; doi:10.1371/journal.pone.0312175)
Supplement: Attachment — Submitted filename: Review Comments_PLOS One.docx [file pone.0312175.s003.docx]

**Meta comments about the article:**

Many thanks to this team for your extremely resonant, meaningful contribution to the literature surrounding wide-ranging impact of music listening for patients and their families in ICU contexts. I particularly appreciate the way your work emphasizes patient perspectives and lived experiences, which both amplifies the patient’s voice in meaningful ways while simultaneously developing much-needed theory surrounding mechanisms of MBI application in the ICU.

The interview quotes you selected were evocative, especially those discussing the complexities of being hospitalized (e.g., grief, restraint, dehumanization), and I thought these were very thoughtfully positioned in tandem with reflections about how music listening impacts patients and their family members across many domains. I also applaud your willingness to push back against the status quo in terms of the types of symptoms that are “typically” studied as outcome measures in research surrounding MBI (e.g., pain, anxiety), allowing patients and their families to take control of the narrative and provide direction for future research.

On a personal note, the impact of music described as described by the patients in this project more fully aligns with my experiences of sharing music with patients in ICUs than those studies which focus on pain and anxiety. These patient perspectives speak to the nuance of music as a holistic, embodied experience, and as something reaches beyond symptom mitigation alone. I appreciate the ways in which your work sits in this space of holding music as a both/and experience.

I offer a small number of reflection points below, which reflect my own curiosities about your work and potential opportunities for expansion/clarification.

**Introduction**

**Definition of MBI:** I noted no mention of music therapy within the manuscript, and wonder if it might make sense to add parentheses following discussion of specialized training (music therapy) as one such example of specialized training related to MBI delivery.

**Limitations of previous research:**

I want to thank the authors for citing limitations of previous research (especially lack of participant diversity which therein results in more diverse music selection), and in particular, your early emphasis on the importance of personally salient music choices rather than provider-selected or standardized music. However, I do feel some additional context may be needed re: why previous studies have used less stimulating music (e.g., slower, instrumental), particularly in a neuro ICU context. This feels relevant to me given the constant risk of overstimulation and lack of ability for an unconscious patient to consent or verbalize felt experiences while music is played. As written, this statement appears dismissive of these choices to control music selection when there are, in reality, complex clinical and sensory factors at play.

Diversity: I will discuss this in greater detail below, but wonder if you might specify what you mean by diversity when referencing previous studies.

**Methods**

I found it interesting that the convenience sample included controlled music listening (e.g., folx who participated in your pilot MBI study) AND uncontrolled music listening as reported by physicians/staff who observed music listening taking place. I assume these are two very different kinds of music listening experiences (e.g., participants in a research project versus participants self-motivated to listen to music of their own accord) and wonder how, if at all, this impacts your findings.

You mention a diverse sample again without clear description/definition of your team’s understanding of a diverse sample (e.g., Diversity of race? Cultural location? Age? Medical status/disability? Gender identity? Music listening preferences? Etc). While I acknowledge that you describe this somewhat in your description of study demographics in the results, I believe a more clear definition, especially at first mention of lack of participant diversity as a prior study limitation, would be meaningful and further contextualize the novel nature of your work.

**Results**

Because patients AND family members participated in this project, I’m curious how your team grappled with the differences in these lived experiences during coding – particularly given that if *only* a family member was interviewed, they cannot actually speak to the patient’s lived experience (only their lived experience of witnessing what the patient is experiencing). This was not cited as a limitation later in your manuscript and is also not captured in your article title (“A qualitative analysis of *patient* experiences”). I suggest amending the title to “patient and family experiences” to be more descriptive of your participant group, and to consider adding a small portion of discussion surrounding the benefits and limitations of equating patient and family experiences as evidence of the same lived experience – as, in my opinion, they are not.

I am also reflecting on the single interview that was conducted 7 months following hospitalization, and the decision to include that interview despite the time length following experiences of active music listening during hospitalization – and, subsequently, the shift in perspective that tends to come following an inpatient stay.

I really appreciate that you provide information surrounding each family’s listening preferences. I know this was not controlled, but it would be very interesting to know how often (on average) each participant listened to music, if this changed along with shifts in a patient’s medical status or a family member’s availability, and whether there were times when music listening was not helpful or even contraindicated as understood by the patients/families themselves.

Small note under the themes subheading: In the third sentence, you indicate that *patients* described the following themes, so it is unclear if these themes come *only* from interviews held directly with patients OR from interviews family members as well. I would advise that the team comb through the manuscript for potential instances of this to clarify patients *and* families where relevant.

In table 3, I’m curious about the use of the word “symptoms” in the title. As I reflect upon the words in this table, they feel to me more like lived/felt experiences of hospitalization and/or emotions/mood states ameliorated or modulated by music listening.

Maintaining cognition, final paragraph: The quote that was selected seems in reference to the patient regaining cognitive abilities more generally, and I’m not as connected to how *music* impacted this patient via this particular quote. Just something to consider.

The humanizing theme was so beautifully written, and deeply resonated with me. “Music reinserted personhood into the clinical space.” Yes! The story about clinicians taking the time to look at photos of a patient then selecting music in response was also powerful to sit with.

I also appreciated the way that you alluded to the potential for music to evoke more challenging memories/feelings. I was hoping this would be explicated more in the discussion, and lead to potential discussion of how this might be negotiated in order to ensure patient and family safety.

Quote about “ancestral plan” - is this a typo/should this read “plane”?

In considering the comment above about potentials of music to evoke challenging feelings, this also feels relevant given your discussions of grief and loss. I think it may be valuable at some point to mention that this presents an opportunity for collaboration (e.g., with psychosocial providers like psychologists, social workers, and music therapists, who are uniquely trained to deliver MBI paired with counseling) to more deeply address grief and trauma-related needs.

Neuro cognitive rehab: While I agree that silence carries just as much risk as noise, overstimulation is also a very real challenge to navigate when implementing music in the ICU, especially with an unconscious patient. It may be worth mentioning here that there are existing (and continually emerging) best practice guidelines for use of music with patients with disorders of consciousness (see this recent chapter from Ferrin & Magee: <https://link.springer.com/chapter/10.1007/978-3-031-50563-8_6>). I caution the authors against making an either/or argument for silence versus sound, but rather, suggest offering a more nuanced perspective that both positive AND negative health outcomes may result from both conditions, and that this is an individualized experience.

Appendix: The interview guide (Question 1) seems to imply that interviews took place DURING active music listening in the hospital (for all but 1 participant, who the team notes was interviewed 7 months later) e.g., “Tell how you came to choose the music we’re playing.” If this is true, I believe this should be clearly stated in the manuscript when describing the interview process as I wonder how the presence of personally salient music playing during an interview about the music would impact patient responses given the evocative nature of music. I’m really curious about this and would love the team to elaborate more!

I also note that the interview guide appears to be very family member focused and am curious how the questions were adapted when interviewing patients directly. There is also a question (11) that alludes to a pre-designed playlist, but the team did not describe specific playlist development and design in the manuscript if this is the case for all participants.
