# Supplementary material for: Personalized music for cognitive and psychological symptom management during mechanical ventilation in critical care: A qualitative analysis
Source: PLoS One. 2024 Oct 24;19(10):e0312175. doi: 10.1371/journal.pone.0312175 (PMC11500878; doi:10.1371/journal.pone.0312175)
Supplement: Attachment — Submitted filename: Response to Reviewers PONE-D-24-19043.docx [file pone.0312175.s004.docx]

Dear Dr. Wolf and Reviewers,

Thank you for the supportive and helpful review of our paper: Personalized music for cognitive and psychological symptom management during mechanical ventilation in critical care: A qualitative analysis. It is wonderful to hear that the stories and results resonated with the reviewers. We found it difficult to choose from the quotes as the participants were incredibly forthcoming in rich and detailed ways. We appreciate the careful reads you have given this paper and the suggestions you have made that will improve this manuscript. Please find attached our responses to your feedback including clarifications, explanations and a summary of changes. Also please note we have changed the title (slightly) to address an area of concern.

*2. Has the statistical analysis been performed appropriately and rigorously?*

*Reviewer #1: Yes*

*Reviewer #2: I Don't Know*

RESPONSE: This is a qualitative study only; statistical analyses are not typically a part of qualitative studies. Therefore, we do not include a statistical analysis for the data presented in this paper.

*3. Have the authors made all data underlying the findings in their manuscript fully available?*

Reviewer #1: Yes

Reviewer #2: No

RESPONSE: There are restrictions on publicly sharing the human research participant data for this study due to participant privacy outlined in our IRB (we do not have permission to share the de-identified data in a repository). Further, the small number of deeply personal interviews contain highly sensitive material, collected from vulnerable people, during a specific period of time and cannot be shared publicly due to risks that participants may be able to be identified through indirect means. In order to maintain confidentiality and compliance with our IRB and to meet our ethical obligations and the data sharing requirements for publication in PLOS, we are amending our Data Statement to include a third-party (tbd) who can manage data requests on a case-by-case basis on our behalf. We have reached out to our IRB and institutional data management leadership to identify an appropriate agent. In the meantime, the authors are happy to field requests as they may arise.

*4. Is the manuscript presented in an intelligible fashion and written in standard English?

PLOS ONE does not copyedit accepted manuscripts, so the language in submitted articles must be clear, correct, and unambiguous. Any typographical or grammatical errors should be corrected at revision, so please note any specific errors here.*

*Reviewer #1: Yes*

*Reviewer #2: No*

RESPONSE: All authors reviewed the manuscript for any unclear, incorrect, or ambiguous language, and we did not identify any sentences that needed improvement. Please identify specific instances, and we are open to making the manuscript more intelligible.

REVIWER: *In the methodology section: the introduction of the method contains information that should be in the previous section. Damasio, Nightingale...These are data from the introduction and not from the methodology section.*

RESPONSE: We moved the description of the theoretical frameworks into a subsection of the Introduction, under the subheading ‘**Theoretical Framework’**.

REVIEWER: *In the methodology section: with what scales did you measure the level of consciousness? When they say devices near the bed, in unconscious patients? (it is not clear to me). What happens with external noise such as mechanical ventilator alarms, infusion pumps, and other confounding variables such as aspiration of secretions, tolerance to orotracheal intubation? Did the patients wake up with the music playing? Or did they only have it while they were asleep/awake?*

RESPONSE: This was a qualitative study only of the views of patients and families who listened to personally relevant music while mechanically ventilated at some point during their ICU stay.

We did not collect measures of level of consciousness during this study. Patients were of varied and changing levels of consciousness during their ICU stay for a variety of reasons (TBI, sedation). Similarly, patients listened to music during a variety of states of consciousness throughout their ICU stay.

In general, speakers and tablets were placed near (within </= 5 feet) for un/semi-conscious patients. After people regained consciousness, we offered them a choice of headphones or speakers/devices. This is described in the section ‘Recorded music listening.’

Because this was a qualitative analysis of an uncontrolled music listening experience, external noises (suctioning, aspiration, etc) were not viewed as confounding variables. Rather, these were all part of the overall experience of being in the ICU on a ventilator.

Because we did not observe all music listening experiences we cannot report on the number of patients who were listening to music while asleep, sedated or in a coma state, nor can we report the number of patients who woke up while listening to music, or who listened to music once awake.

REVIEWER: *In the results section: Excellent presentation of the qualitative results.
But regarding the quantitative results, I have lacked the ability to know times*

*they listening to music, how many times?, according to each patient?*

RESPONSE: This was a qualitative study only. (This was **not** the qualitative of a quantitative study). For this analysis we did not perform any measurements, instead we report the views of patients and their families, gathered through interviews and conversations during and after ICU hospitalization.

We did not use a standardized protocol for music listening in this study. Our study focuses on querying the experiences of music listening after the fact. To make this clearer, we removed the words ‘use of’ from the header which now reads **Recorded Music Listening**

We did not collect information about how often or when patients listened to music or how the listening practices changed during the ICU stay. We added these points as limitations to our study.

In the limitations: **First, we do not know how often or when (times of day) patients listened to music or if preferred music changed alongside shifts in clinical conditions**.

REVIEWER: *What levels of consciousness did they have depending on the moment they were listening to the music? There are 14 patients. I think they could improve table 2, showing this data.*

RESPONSE: We did not measure level of consciousness during this study. As we describe above, patients experienced a range of levels of consciousness throughout the ICU hospitalization and listened to music during a spectrum of states of consciousness. We are reporting the patient and family descriptions of these experiences in this paper.

We conducted 14 interviews for this analysis, but the data used to develop the themes also includes notes from several other people (patients, families, clinicians) who used music during the hospitalization. We made reference to Appendix 2 in the original submission, but neglected to include it. Some of the other informants are now listed in Appendix 2.

**Appendix 2: Other Informants**

**Parent of young man with coma after a motor vehicle crash (MVC)**

**Parent of young man with coma after pedestrian versus automobile (PVA)**

**Partner of young man with traumatic brain injury (TBI) after bicycle crash**

**Sibling of middle-aged man with cerebral vascular accident (CVA) and coma after MVC**

**Middle aged man with blunt chest trauma**

**Bedside registered nurses**

**Sibling of young woman with TBI after MVC**

**Medical doctors of neurology, critical care, surgery and neurosurgery: professors, and trainees**

**Spouse and sister-in-law of man with TBI**

**Middle aged woman after abdominal surgery**

**Middle aged man with abdominal injuries**

**Family of older woman after CVA**

**Advanced Practice Providers on the trauma service**

**Uncle and parent of young man with assault related injuries**

**Parents and sibling of young man with assault related injuries and TBI**

**Siblings of young man with assault related injuries**

REVIEWER: ***Introduction Definition of MBI****:* *I noted no mention of music therapy within the manuscript, and wonder if it might make sense to add parentheses following discussion of specialized training (music therapy) as one such example of specialized training related to MBI delivery.*

RESPONSE: We amended the language in the text to be:

When music is used in the clinical setting to achieve a health-related goal such as symptom management, it is called a music-based intervention (MBI) **[12]. MBIs may be delivered by a credentialled music therapist with specialized training (music therapy) or by a health care provider (music medicine) [13, 14].**

REVIEWER: ***Limitations of previous research:*** *I want to thank the authors for citing limitations of previous research (especially lack of participant diversity which therein results in more diverse music selection), and in particular, your early emphasis on the importance of personally salient music choices rather than provider-selected or standardized music. However, I do feel some additional context may be needed re: why previous studies have used less stimulating music (e.g., slower, instrumental), particularly in a neuro ICU context. This feels relevant to me given the constant risk of overstimulation and lack of ability for an unconscious patient to consent or verbalize felt experiences while music is played. As written, this statement appears dismissive of these choices to control music selection when there are, in reality, complex clinical and sensory factors at play.*

RESPONSE: The practice in our ICU (including in the neuroscience ICU, guided by the medical directors of neurosurgery and neurocritical care) has been to encourage the most familiar forms of cognitive stimulation with an overall aim to try to awaken people from unconsciousness/coma, and to observe patients carefully for their tolerance of this (vital signs, facial expressions). This fits with movements in the critical care community to avoid sedation and to encourage awake ICUs. Saying that, we acknowledge the lack of evidence exploring the use of uncontrolled music selection, and the value or safety of stimulating music for unconscious/comatose patients. The topic of controlled music selection (especially as it relates to tempo, mood, language) merits further consideration and is an area of interest for our group for future research.

We removed the language: “**to slow tempo instrumental pieces**” to avoid oversimplifying the matter, and we have changed the words “**ignores**” to “**may contravene**” to be less dismissive.

REVIEWER*: Diversity: I will discuss this in greater detail below, but wonder if you might specify what you mean by diversity when referencing previous studies.*

RESPONSE: We added ‘socio-cultural’ in this portion of the text to be concise and are more detailed later in the methods:

“Lack of participant **socio-cultural** diversity is another important limitation in North American studies of MBI in MV adults”.

REVIEWER: ***Methods*** *I found it interesting that the convenience sample included controlled music listening (e.g., folx who participated in your pilot MBI study) AND uncontrolled music listening as reported by physicians/staff who observed music listening taking place. I assume these are two very different kinds of music listening experiences (e.g., participants in a research project versus participants self-motivated to listen to music of their own accord) and wonder how, if at all, this impacts your findings.*

RESPONSE: We may have created some confusion through the use of the words ‘pilot study’. The ‘pilot’ was simply to develop and implement a music listening program and see how people responded to it; there were no ‘controlled music listening’ sessions for this study. Most, but not all, of the patients listened to music chosen by their families when they were un/semi-conscious or sedated, and chose their own music as they regained consciousness. As you point out, there is likely a real difference in the music listening experience when a person is semi/unconscious (akin to a controlled listening experience) and when it is patient-initiated. We removed reference to the pilot study to clarify this and will address these differences more carefully in the limitations/discussion.

REVIEWER: *You mention a diverse sample again without clear description/definition of your team’s* *understanding of a diverse sample (e.g., Diversity of race? Cultural location? Age? Medical status/disability? Gender identity? Music listening preferences? Etc). While I acknowledge that you describe this somewhat in your description of study demographics in the results, I believe a more clear definition, especially at first mention of lack of participant diversity as a prior study limitation, would be meaningful and further contextualize the novel nature of your work.*

RESPONSE: We added language to clarify what is intended for a ‘diverse’ sample. See below:

Purposive sampling was used to ensure a **socially, culturally, ethnically, linguistically, and economically** diverse sample of respondents and was ongoing until thematic saturation was achieved.

***REVIEWER: Results*** *Because patients AND family members participated in this project, I’m curious how your team grappled with the differences in these lived experiences during coding – particularly given that if only a family member was interviewed, they cannot actually speak to the patient’s lived experience (only their lived experience of witnessing what the patient is experiencing). This was not cited as a limitation later in your manuscript and is also not captured in your article title (“A qualitative analysis of patient experiences”). I suggest amending the title to “patient and family experiences” to be more descriptive of your participant group, and to consider adding a small portion of discussion surrounding the benefits and limitations of equating patient and family experiences as evidence of the same lived experience – as, in my opinion, they are not.*

RESPONSE: Thank you for raising this important question. We thought about this a lot and acknowledge that these perspectives are not the same. As you noted there were 3 interviews of family members only, and the other 11 were either of patients alone, or of patients with their family members present. We wanted to make sure that patients who could not [ever] speak for themselves still had a voice and that some part of their experience was incorporated into the analysis. Including their physical gestures was one way, but this too involved a certain level of interpretation (often by clinicians). Rather than ‘trust’ ourselves, we thought it better to look to families to help us understand what they felt their loved one was going through. We were [somewhat] assured that these views were a reliable representation after reflecting on data from prior studies that suggests that family/friend assessment of symptoms are highly correlated with patients’ self-report (https://pubmed.ncbi.nlm.nih.gov/22890258/).

We decided not to call these ‘family-experiences’ of music listening since they were views of families of the *patient’s experiences* (not their own experiences of the music listening program).

As an extra layer of caution, we coded all family-member perspectives for their views of the patients’ experiences and used these codes to confirm and illustrate concepts identified in the patient interviews. We added some language to the methods section to make this clearer and also some to the discussion.

**‘Family-member perspectives were coded for their views of the patients’ experience and were used to confirm and illustrate themes and concepts identified in the patient interviews.’**

Regarding the title of the manuscript. We acknowledge that some of our findings are indeed adjacent to a ‘lived experience’. However, because we used a Grounded Theory methodology (and not Phenomenology) to frame our interviews, data collection, coding and analysis, we have positioned this paper around the social process about what is occurring: the why, what for, how, about personally relevant music listening. We have used this to learn about how people use personally relevant music listening, how it works and what people think about it in general. The lived experience of music listening in critical care is definitely an area for future exploration something we would want to do once a program was more established. To make this clearer, we changed the title, removing the language of ‘experiences’. It now reads:

**Personalized music for cognitive and psychological symptom management during mechanical ventilation in critical care: A qualitative analysis.**

*REVIEWER: I am also reflecting on the single interview that was conducted 7 months following hospitalization, and the decision to include that interview despite the time length following experiences of active music listening during hospitalization – and, subsequently, the shift in perspective that tends to come following an inpatient stay.*

RESPONSE: We looked carefully at this interview and considered whether or not to include it for the reasons you mention. However, the views expressed were consistent with those conducted closer to the ICU stay especially as they related to sparking the mind into action, regaining consciousness, awareness, and humanizing the ICU. We think that it is helpful to hear how stable and similar these ideas were across time.

*REVIEWER: I really appreciate that you provide information surrounding each family’s listening preferences. I know this was not controlled, but it would be very interesting to know how often (on average) each participant listened to music, if this changed along with shifts in a patient’s medical status or a family member’s availability, and whether there were times when music listening was not helpful or even contraindicated as understood by the patients/families themselves.*

RESPONSE: We added some language to the limitations to address how little we know about the music listening itself. There were no moments when families or patients felt that music listening was not helpful/contraindicated. In fact many times families and patients complained that they did not feel there was ‘enough music’. For most the concept was so novel, that they had not formed an idea about best moments for it. The specifics of exactly when and how were outside the scope of this analysis, and merit further study, though we would recommend performing this sort of analysis after a music listening program is better established to make sure that the views are rooted in family and patient perceptions, and not unduly influenced by staff uncertainty, lack of equipment or lack of protocol (or pandemic restrictions).

There are several limitations to this study. **First, we don’t know how often or when (times of day) patients listened to music or if preferred music changed alongside shifts in clinical conditions. Nor do we know how well aligned the family chosen music was with patient choice once able to engage. The reliability of surrogate selection of personally relevant music for unconscious people has yet to be established. Another limitation** is that all patients in this study identified as men.

REVIEWER: *Small note under the themes subheading: In the third sentence, you indicate that patients described the following themes, so it is unclear if these themes come *only* from interviews held directly with patients OR from interviews family members as well. I would advise that the team comb through the manuscript for potential instances of this to clarify patients and families where relevant.*

RESPONSE**:** We reviewed the manuscript and made changes to make sure that patients’ views and families’ views are carefully labeled as such and only combined when shared. In many instances this involved replacing the word ‘participant’ to be specific about whom.

*REVIEWER: In table 3, I’m curious about the use of the word “symptoms” in the title. As I reflect upon the words in this table, they feel to me more like lived/felt experiences of hospitalization and/or emotions/mood states ameliorated or modulated by music listening.*

RESPONSE: We added the word ‘Experiences’ to the table title such that it now reads:

**Table 3: Symptoms and Experiences affected by Personalized Music Listening in Critical Care**

*REVIEWER: Maintaining cognition, final paragraph: The quote that was selected seems in reference to the patient regaining cognitive abilities more generally, and I’m not as connected to how music impacted this patient via this particular quote. Just something to consider.*

RESPONSE: We added some language to clarify this moment (a lovely moment when the patient was being very particular about a point of view, strictly analyzing a discussion point during an interview at a high level of detail, his mother witnessed this as she was present for the interview and attributed his agility to having listened to music when he was on the ventilator.

‘In another case, a mother **was amazed by her adult son’s preserved linguistic and cognitive agility, after witnessing his ability** to construct careful arguments despite having a TBI. She attributed this to his **having listened to music when he was recovering on the ventilator**.’

REVIEWER*: The humanizing theme was so beautifully written, and deeply resonated with me. “Music reinserted personhood into the clinical space.” Yes! The story about clinicians taking the time to look at photos of a patient then selecting music in response was also powerful to sit with.*

RESPONSE: Thank you for saying so. This moment between the staff, the patient and their family was massive. It felt good (and true to the piece) to be able to tell others about it.

*REVIEWER: I also appreciated the way that you alluded to the potential for music to evoke more challenging memories/feelings. I was hoping this would be explicated more in the discussion, and lead to potential discussion of how this might be negotiated in order to ensure patient and family safety.*

RESPONSE: We added some language in the discussion to open that up a little further. See below.

REVIEWER*: Quote about “ancestral plan” - is this a typo/should this read “plane”?*

RESPONSE: We returned to the recordings, listened to this segment and can confirm that the speaker said ‘ancestral plan’.

REVIEWER: *In considering the comment above about potentials of music to evoke challenging feelings, this also feels relevant given your discussions of grief and loss. I think it may be valuable at some point to mention that this presents an opportunity for collaboration (e.g., with psychosocial providers like psychologists, social workers, and music therapists, who are uniquely trained to deliver MBI paired with counseling) to more deeply address grief and trauma-related needs.*

RESPONSE: *We added the following language to the manuscript.* ***Playing personally relevant music represents an opportunity for collaboration with psychosocial providers (e.g. music therapists, violent injury prevention professionals, social workers, chaplains and psychologists) to more deeply address grief and trauma-related needs.***

REVIEWER: *Neuro cognitive rehab: While I agree that silence carries just as much risk as noise, overstimulation is also a very real challenge to navigate when implementing music in the ICU, especially with an unconscious patient. It may be worth mentioning here that there are existing (and continually emerging) best practice guidelines for use of music with patients with disorders of consciousness (see this recent chapter from Ferrin & Magee:* [*https://link.springer.com/chapter/10.1007/978-3-031-50563-8_6*](https://link.springer.com/chapter/10.1007/978-3-031-50563-8_6)*). I caution the authors against making an either/or argument for silence versus sound, but rather, suggest offering a more nuanced perspective that both positive AND negative health outcomes may result from both conditions, and that this is an individualized experience.*

RESPONSE: Thank you for the attached reference which we found most helpful. We added a sentence to include the value of rest (and referenced the guidelines you recommend above).

**Indeed, current best practice guidelines for music listening during recovery from disordered consciousness favor a mix of salient music listening with periods of rest [53].**

REVIEWER: *Appendix: The interview guide (Question 1) seems to imply that interviews took place DURING active music listening in the hospital (for all but 1 participant, who the team notes was interviewed 7 months later) e.g., “Tell how you came to choose the music we’re playing.” If this is true, I believe this should be clearly stated in the manuscript when describing the interview process as I wonder how the presence of personally salient music playing during an interview about the music would impact patient responses given the evocative nature of music. I’m really curious about this and would love the team to elaborate more!*

RESPONSE: None of the interviews were conducted during active music listening. We amended the language of the interview guide to be clearer. It should be noted that this was a *guide only*; the interviews were very fluid. In most cases patients and their families just started talking and answered many of the concepts we were curious about without being asked directly.

REVIEWER: *I also note that the interview guide appears to be very family member focused and am curious how the questions were adapted when interviewing patients directly. There is also a question (11) that alludes to a pre-designed playlist, but the team did not describe specific playlist development and design in the manuscript if this is the case for all participants.*

RESPONSE: Here too, the language of the interview guide as presented was not clear. We amended it to be more clearly focused on asking questions of patients (along with their families). Again, we offer this guide as an example of how we started the process of data collection/interviews, and note that we amended it throughout the study period depending on the usefulness of some questions, and the responses we were starting to see.

**ADDED REFERENCES**: Note that we have added 6 references to this manuscript.

**Reference number 13 and 14** used in the following sentence: *MBIs may be delivered by a credentialled music therapist with specialized training (music therapy) or by a health care provider (music medicine) [13, 14].*

#13 Stegemann T, Geretsegger M, Phan Quoc E, Riedl H, Smetana M. Music Therapy and Other Music-Based Interventions in Pediatric Health Care: An Overview. Medicines (Basel, Switzerland). 2019;6(1):25.

#14 Sihvonen AJ, Särkämö T, Leo V, Tervaniemi M, Altenmüller E, Soinila S. Music-based interventions in neurological rehabilitation. The Lancet Neurology. 2017;16(8):648-60

**Reference 23**, used in the following sentence: *First, despite recommendations to use patient preferred music for symptom management in critical care [22], most studies of MBI in MV adults use music selected by investigators or limit patient choice [23].*

#23 Menza R, Bongiovanni T, Leutwyler H, Tang J, Johnson JK, Howie-Esquivel J. Music-Based Interventions for Symptom Management in Critically Ill, Mechanically Ventilated Adults: A Scoping Review of the Literature. Journal of integrative and complementary medicine. 2024.

**Reference 34**, used in the following sentence: *This study adhered to the COREQ guidelines for reporting qualitative research [34].*

#34 Tong A, Sainsbury P, Craig J. Consolidated criteria for reporting qualitative research (COREQ): a 32-item checklist for interviews and focus groups. International Journal for Quality in Health Care. 2007;19(6):349-57

**Reference 35**, used in the following sentence: All interview transcripts, summaries and field notes were entered into Atlas.ti 9.1[35].

\# 35 ATLAS.ti Scientific Software Development GmbH. ATLAS.ti V. 9.1.2 ATLAS.ti Scientific Software Development GmbH. 2021

**Reference 55**, used in the following sentence: *Indeed, current best practice guidelines for music listening during recovery from disordered consciousness favor a mix of salient music listening with periods of rest [55].*

#55 Perrin F, Magee WL. Music as Sensory Stimulation and Therapeutic Intervention. In: Schnakers C, Laureys S, editors. Coma and Disorders of Consciousness. Cham: Springer International Publishing; 2023. p. 99-113.
